# Advancing care for women and girls with haemophilia: evidence from a comprehensive longitudinal analysis

**DOI:** 10.1016/j.rpth.2025.103232

**Published:** 2025-10-22

**Authors:** Wolfgang Miesbach

**Affiliations:** Medical Clinic 2, Institute of Transfusion Medicine, University Hospital Frankfurt, Frankfurt, Germany

Hemophilia, caused by mutations in the F8 and F9 genes that result in deficiencies of coagulation factor (F)VIII and FIX, respectively, has undergone a remarkable transformation through the development of factor replacement therapy, subcutaneous treatments, and comprehensive prophylactic strategies [1]. These advances have not extended equitably to all populations, particularly women and girls with bleeding disorders.

The marginalization of women's bleeding symptoms in hemophilia spans over 2 centuries [2,3]. Therapeutic innovations and clinical research in hemophilia have predominantly focused on male patients, leaving women and girls frequently excluded from these advancements. Female hemophilia carriers (HCs)—those who inherit 1 abnormal X chromosome—may nonetheless exhibit bleeding symptoms. This occurs through X chromosome inactivation mechanisms, which can significantly reduce their factor levels even when a normal gene copy is present [4].

While systematic recognition began around 1990, substantial barriers persist. Healthcare disparities affecting HCs include diagnostic delays averaging 7 years longer than those of males with comparable factor levels, inappropriate clinical interventions, and higher rates of complications, including anemia and surgical procedures [5,6]. A study published in August 2025 examined the lived experiences of 28 Dutch women with congenital bleeding disorders aged 18 to 40 years, revealing significant gaps in healthcare knowledge and support [7]. The research identified that healthcare providers outside specialized hemophilia treatment centers frequently lacked knowledge about bleeding disorders in women and consistently downplayed symptoms, particularly heavy menstruation, leading to diagnostic delays and inadequate care. The study found limited awareness among healthcare professionals regarding the substantial mental health impacts these conditions have on women's daily lives, especially during critical life transitions such as adolescence and the move to higher education. These findings highlight the urgent need for improved healthcare provider education and more comprehensive, life-stage appropriate support systems for women with these conditions.

Recently, international guidelines from the International Society on Thrombosis and Haemostasis (ISTH), the European Association for Haemophilia and Allied Disorders/European Haemophilia Consortium, and the National Bleeding Disorders Foundation Medical and Scientific Advisory Council (MASAC) have established comprehensive frameworks for women's care (Table). The 2021 ISTH nomenclature expanded and refined the terminology, establishing 5 evidence-based categories based on factor activity levels and bleeding phenotype [8]. European principles (European Association for Haemophilia and Allied Disorders/European Haemophilia Consortium) emphasize systematic screening, timely diagnosis, and multidisciplinary care approaches [9]. The 2024 MASAC Document 286 provides the most comprehensive update on the diagnosis and management of inherited bleeding disorders in girls and women, mandating iron deficiency screening, even with normal hemoglobin levels, and addressing historical underdiagnosis gaps [10]. Despite these developments, implementation gaps remain significant, with the persistent 7-year diagnostic delay continuing to disadvantage female patients across healthcare systems [5].

**Table:** International guidelines for women and girls with bleeding disorders.

Organization, date	Content	Impact
ISTH, 2021 [8]	• Eliminated outdated "carrier" terminology • Established 5 evidence-based categories:- Severe (<0.01 IU/mL)- Moderate (0.01-0.05 IU/mL)- Mild (0.05-0.40 IU/mL)- Symptomatic (≥0.40 IU/mL with bleeding)- Asymptomatic (≥0.40 IU/mL without bleeding)	Improved diagnostic accuracy and research criteria; enhanced clinical care for previously underrecognized populations
EAHAD and EHC, 2021 [9]	• Established 10 comprehensive care principles • Enhanced quality assurance across European centers • Equitable access to care • Systematic screening implementation • Timely diagnosis • Multidisciplinary care approach • Family-centered care • Specialized gynecological care	Promoted systematic screening across European centers; a framework for standardized care delivery
NBDF MASAC, 2024 [10]	• Enhanced bleeding assessment tools • Mandated iron deficiency screening (ferritin < 30 ng/mL) • Reduced diagnostic delays even with normal Hb • Emphasized multidisciplinary care approach • Addressed historical underdiagnosis gaps	Addresses historical underdiagnosis gaps; improved iron deficiency detection; enhanced quality of care standards

EAHAD, European Association for Haemophilia and Allied Disorders; EHC, European Haemophilia Consortium; Hb, hemoglobin; ISTH, International Society on Thrombosis and Haemostasis; IU, International Units; MASAC, Medical and Scientific Advisory Council; NBDF, National Bleeding Disorders Foundation.

The study by Swaminathan et al. [11] represents the most comprehensive analysis of bleeding events and treatment utilization across the entire lifespan of HCs. This analysis of 3663 female carriers (2728 with hemophilia A and 935 with hemophilia B) from 2010 to 2020 employed a 3-tier age stratification (0-12, 13-49, and >50 years), extending the analysis beyond traditional reproductive-focused studies. This represents the first systematic investigation to longitudinally track both coagulation factor levels and bleeding symptoms over time, providing insights into the temporal evolution of both laboratory and clinical parameters in HCs. Joint bleeding across all age groups proved particularly striking, with an increasing incidence in older carriers: 20% (0-12 years), 29% (13-49 years), and 38% (>50 years) for hemophilia A carriers. This challenges the traditional notion that joint bleeding primarily concerns the reproductive years and highlights the need for lifelong musculoskeletal surveillance in carriers. Recent ultrasound imaging studies confirm subclinical joint changes even in carriers with normal factor levels [12], supporting the need for longitudinal orthopedic monitoring. Compared with age-matched controls, HCs demonstrate significantly poorer outcomes: 50% report chronic joint pain vs 3.3% of controls, with bleeding symptoms occurring irrespective of baseline factor activity levels [6]. These findings parallel patterns observed in males with mild hemophilia not receiving prophylaxis, suggesting that cumulative microbleeds contribute to progressive joint disease [13].

The relationship between factor levels and bleeding phenotype proved more complex than anticipated. Most HCs had factor levels near the normal range (57.1% of hemophilia A carriers and 48.2% of hemophilia B carriers had levels > 40%), yet bleeding remained more frequent among hemophilia A carriers than among hemophilia B carriers [11], reinforcing previous observations that factor levels alone inadequately predict bleeding risk [12]. A novel finding was the significant age-related increase in FVIII levels in hemophilia A carriers (*P* < .001) [11]. This phenomenon likely reflects the well-documented rise in von Willebrand factor levels with ageing, as von Willebrand factor serves as FVIII’s carrier protein [14]. The clinical significance of this phenomenon remains unclear—whether it provides protective benefits against bleeding or reduces perioperative risks requires a prospective study.

The observed increase in both factor concentrate and nonfactor hemostatic therapy utilization from 2010 to 2020 reflects the growing clinical awareness of bleeding risks in carriers. Hemophilia B carriers received factor therapy more frequently than hemophilia A carriers (33% vs 22.7%) [11], likely reflecting limited alternative treatments, such as desmopressin, for FIX deficiency. This trend likely predates the 2021 MASAC recommendations for factor replacement prophylaxis in carriers with factor levels < 50% prior to delivery, suggesting that clinician recognition of carrier bleeding risks has been evolving independently of formal guidelines [10]. Fewer than 12% of carriers use medical alert devices, representing a significant patient safety concern [5]. This low utilization rate reflects persistent misconceptions about carrier bleeding risks and highlights the need for enhanced patient education. Medical alert devices are particularly crucial for carriers, as emergency healthcare providers may not recognize hemophilia carriership as a bleeding disorder, potentially leading to inadequate hemostatic management during emergency procedures. A 2024 study found that only 18% of females who were predicted to need care had accessed hemophilia treatment centers in well-resourced countries [2].

These findings necessitate fundamental changes in clinical practice:

• Comprehensive risk assessment: recognition that factor levels alone inadequately predict bleeding risk in HCs, requiring systematic evaluation of bleeding phenotype and family history.
• Lifelong joint surveillance: implementation of routine joint health monitoring throughout the lifespan, extending beyond reproductive years, with consideration of prophylactic interventions to prevent progressive arthropathy.
• Education: targeted training for healthcare professionals in and outside hemophilia treatment centers to early recognize bleeding symptoms and avoid inappropriate symptom minimization.
• Inclusive clinical research: systematic incorporation of women and girls in clinical trials for emerging therapies, including extended half-life factors, nonfactor therapies, and gene therapy products.
• Female-specific outcomes: development of clinical trials designed specifically to evaluate female bleeding patterns and treatment responses.

The comprehensive data presented by Swaminathan et al. [11] provide compelling evidence that HCs face significant bleeding risks throughout their lifespan, challenging traditional perceptions and demanding evidence-based practice changes [5]. Only through the systematic implementation of current guidelines, enhanced safety measures, and continued research—including across diverse populations—can true equity in bleeding disorder care be achieved.
